# Functional Diversity of Fungal Communities in Soil Contaminated with Diesel Oil

**DOI:** 10.3389/fmicb.2017.01862

**Published:** 2017-09-27

**Authors:** Agata Borowik, Jadwiga Wyszkowska, Karolina Oszust

**Affiliations:** ^1^Department of Microbiology, University of Warmia and Mazury in Olsztyn, Olsztyn, Poland; ^2^Institute of Agrophysics, Polish Academy of Sciences, Lublin, Poland

**Keywords:** fungal communities, functional diversity, diesel oil, pollution, degradation of PAHs, FF PLATE^®^

## Abstract

The widespread use and consumption of crude oil draws the public’s attention to the fate of petroleum hydrocarbons in the environment, as they can permeate the soil environment in an uncontrollable manner. Contamination of soils with petroleum products, including diesel oil (DO), can cause changes in the microbiological soil properties. The effect of diesel oil on the functional diversity of fungi was tested in a model experiment during 270 days. Fungi were isolated from soil and identified. The functional diversity of fungal communities was also determined. Fungi were identified with the MALDI-TOF method, while the functional diversity was determined using FF-plates made by Biolog^®^, with 95 carbon sources. Moreover, the diesel oil degradation dynamics was assessed. The research showed that soil contaminated with diesel oil is characterized by a higher activity of oxireductases and a higher number of fungi than soil not exposed to the pressure of this product. The DO pollution has an adverse effect on the diversity of fungal community. This is proved by significantly lower values of the Average Well-Color Development, substrates Richness (R) and Shannon–Weaver (H) indices at day 270 after contamination. The consequences of DO affecting soil not submitted to remediation are persistent. After 270 days, only 64% of four-ringed, 28% of five-ringed, 21% of 2–3-ringed and 16% of six-ringed PAHs underwent degradation. The lasting effect of DO on communities of fungi led to a decrease in their functional diversity. The assessment of the response of fungi to DO pollution made on the basis of the development of colonies on Petri dishes [Colony Development (CD) and Eco-physiological Diversity (EP) indices] is consistent with the analysis based on the FF MicroPlate system by Biolog^®^. Thus, a combination of the FF MicroPlate system by Biolog^®^ with the simultaneous calculation of CD and EP indices alongside the concurrent determination of the content of PAHs and activity of oxireductases provides an opportunity to achieve relatively complete characterization of the consequences of a long-term impact of diesel oil on soil fungi.

## Introduction

The widespread use of crude oil and petroleum products in the world leads to an increasing contamination of the natural environment (Baran et al., 2004; Albert and Tanee, 2011; Lebrero et al., 2012; Mohsenzadeh et al., 2012; Souza et al., 2014; Nwaichi et al., 2015). One of the underlying reasons is the technical, industrial and economic progress. Each year, around 0.10 to 0.25% of petroleum products pervade the natural environment (Garcia-Lor et al., 2012). According to the International Energy Agency [IEA] (2016), the global demand for crude oil in the first quarter of 2016 was 95.0 mln barrels d^-1^. The consumption of biodiesel oil in the world increases dramatically. Hence, the contamination of the natural environment with products originating from crude oil processing raises concern in both industrial and developing countries (Lebrero et al., 2012). Because petroleum products are a mixture of hydrocarbons with low bioavailability, which are often carcinogenic and mutagenic compounds (Souza et al., 2014), they are considered to be among the most toxic and dangerous pollutants in particular compartments of nature, especially in soil, which is the major pool of their accumulation (Sutton et al., 2013; Covino et al., 2016; Marchand et al., 2017). They can cause changes in of fungal biodiversity of soil. This can have an unfavorable impact on the soil health and grown plants (Alrumman et al., 2015).

The presence of petroleum products in the natural environment is a grave problem, because it causes gradual soil degradation in many parts of the world (Albert and Tanee, 2011), and sometimes leads to the permanent destruction of soil, loss of its fertility and disappearance of the plant cover (Wyszkowska et al., 2015). Petroleum substances have a high potential to accumulate in the soil environment, where they can interfere with the soil’s microbiome (Kucharski et al., 2010; Wyszkowska and Wyszkowski, 2010; Lipińska et al., 2013, 2014a,b; Kaczyńska et al., 2015; Nwaichi et al., 2015; Wyszkowska et al., 2015).

Much importance is attached to fungi in bioremediation of soils contaminated with petroleum products, one reason being their adaptability to extreme conditions in habitat (Schadt et al., 2003; Rousk et al., 2010). In general, fungi demonstrate quite a large range of pH in which they can obtain an optimal growth (Rousk et al., 2010). The biomass of fungi is positively correlated with their diversity (Setälä and McLean, 2004). They respond positively to the input of organic matter to soil (Gomez et al., 2006; Islam et al., 2011; Mohsenzadeh et al., 2012). They can be effective in the removal of petroleum products from soil, e.g., some species of the genera *Aspergillus* (Díaz-Ramírez et al., 2013; El-Hanafy et al., 2017) and *Candida* (Fan et al., 2014; Silva et al., 2015). High capacity to degrade diesel oil is also ascribed to such fungi as *Alternaria alternaria, Aspergillus terreus, Cladosporium sphaerospermum, Eupenicillium hirayamae, Paecilomyces variotii, Trichoderma tomentosum*, and *Fusarium oxysporum* (Ameen et al., 2016; Jiang et al., 2016; Marchand et al., 2017). On the one hand, the fungi present in soil polluted with petroleum products are beneficial for this environment as they contribute to the removal of PAHs from soil. On the other hand, when such fungi appear in fuel tanks, they can add to the deterioration of petroleum products (Bentoa and Gaylardeb, 2001). The ability of fungi to survive in environments contaminated with petroleum products and petroleum fuels alone suggests a reciprocal relationship between these fungi and the products mentioned (Alrumman et al., 2015). Next to bacteria, fungi affect transformations of these products, whereas petroleum products produce some influence on the growth of fungi (Kucharski and Jastrzębska, 2005; Alrumman et al., 2015).

Precise identification of the impact of petroleum products on the natural environment is still difficult. One reason is that our knowledge of the diversity of microorganisms, especially fungi, present in environments contaminated with these products is incomplete and fragmentary (Jiang et al., 2016; Marchand et al., 2017). In such soils, changes may occur in proportions between counts of fast and slow growing microorganisms (Kucharski and Jastrzębska, 2005; Wyszkowska and Kucharski, 2005), and the succession of microorganisms is a necessary condition for effective soil purification from petroleum products. In addition, PAHs contained in petroleum products can be an excellent source of energy for some microorganisms (Sun et al., 2010).

In view of the above, the following study has been undertaken in order to determine the abundance and functional diversity of fungal communities, based on an analysis of the metabolic profile in soil polluted with diesel oil. This problem was scrutinized in the context of the duration impact (from 7 to 270 days) of diesel oil on soil fungal assemblages. Also, the percentage degradation of PAHs by soil microorganisms was determined. The assessment was supported by determinations of the activity of selected enzymes participating in processes of carbon transformation.

## Materials and Methods

### Soil Material

Model tests were performed on soil samples with the textural composition of loamy sand, collected from the arable humic horizon (depth of 0–20 cm) of proper eutrophic brown earths, which were classified according to the World Reference Base of Soil Resources (IUSS Working Group WRB, 2014) as Eutric Cambisols. The soil used for further studies originated from fields used for research purposes and located at the Research Station in Tomaszkowo (NE Poland, 53.7161° N, 20.4167° E), a village in the Olsztyn Lake District. Selected properties of the soil are presented in **Supplementary Table S1**. The physicochemical characteristics of soil were determined with the following methods: the grain-size distribution by the areometric method (PN-R-04032, 1998), pH potentiometrically in 1 Mol KCl dm^-3^ (ISO 10390, 2005), hydrolytic acidity and the sum of exchangeable base cations by the Kappen method (Carter, 1993), content of organic carbon by the Tiurin method, total content of nitrogen by the Kjeldahl method (Nelson and Sommers, 1996), and the content of exchange cations K^+^, Na^+^, Ca^2+^, Mg^2+^ by flame photometry (Harris, 2006).

### Characteristics of Diesel Oil Ekodiesel ULTRA

Ekodiesel Ultra class B, purchased at a PKN ORLEN petrol station, was tested in the experiment. This product is used to power self-ignition engines in road transport. It is popular mostly in large agglomerations and protected nature areas. The density of diesel oil is 820–845 g dm^-3^, while the maximum content of polycyclic aromatic hydrocarbons (PAHs) is 7% (m/m), solid impurities – 24 mg kg^-1^, sulfur – 10 mg kg^-1^. More detailed information about Ekodiesel ULTRA can be found online^1^.

### The Experiment

Having selected soil for the tests, the second stage of the study was conducted under strictly controlled conditions in order to eliminate the impact of variable factors. Dark glass containers, 1.0 dm^3^ in capacity, were each filled with 1 kg of properly prepared, air dry soil, previously passed through a 2 mm mesh sieve. Each batch of soil (1 kg) was applied a single dose of Ekodiesel Ultra B equal 50 cm^3^ per 1 kg d.m. of soil. The soil material was carefully mixed with diesel oil, after which demineralised water was added to achieve 40% of capillary water capacity. The whole set of containers was covered with perforated foil and incubated in a thermostat at 22°C for 270 days. The soil moisture was monitored throughout the whole experiment and any water loss was replenished. The control sample consisted of soil unpolluted with diesel oil. The experiment included three replicates.

### Isolation and Identification of Fungi in Soil

At all dates of analyses (days 7, 30, 60, 90, and 270 of incubation), counts of fungi were determined using the method of dilutions of soil smears for both unpolluted soil and soil contaminated with Ekodiesel Ultra B. Fungi were grown on Martin’s medium (1950) with addition of rose bengal and aureomycin antibiotic addition. Microorganisms were cultured on Petri dishes, with five replicates, at a constant temperature of 28°C. The number of colony forming units (cfu) was determined using a colony counter. In addition, the influence of diesel oil on the structure of fungal communities and their biodiversity was identified. To this aim, appropriate dilutions (10^-4^ and 10^-5^) of a soil solution suspension were transferred in parallel onto Petri dishes, in five replications, and then incubated in an incubator at 28°C. For 10 days, the grown colonies of fungi were counted every day, after which values of the colony development index (CD) and the eco-physiological diversity index (EP) were calculated (**Supplementary Table S2**).

On day 270 of the experiment, fungi were isolated from unpolluted and diesel oil polluted soil. Isolated fungi were inoculated 10 times in order to obtain pure cultures. The isolated fungi were identified with mass spectrometry MALDI-TOF MS, based on matrix-assisted laser desorption/ionization coupled with the use an ion flow tube (**Supplementary Figure S1**). The mass spectrometry method relies on an analysis of the unique protein profile, specific for each species of microorganisms, which is referred to as a molecular “fingerprint” (Wieser et al., 2012). Analysis of the protein profile of a microorganism and its comparison with the reference set of proteins of microorganisms allow us to identify the species of the examined microorganism (Chalupová et al., 2014).

### The Catabolic Profile of Soil Fungal Community Achieved with FF MicroPlates^®^

On the same dates when counts of fungi and activity of dehydrogenases and catalase were determined, i.e., days 7, 30, 60, 90, and 270 of the experiment, the metabolic profile of soils contaminated with diesel oil was determined in three consecutive replications. The metabolic capacities of fungi were determined using the system FF MicroPlate^®^ with 95 carbon sources. Soil suspension for the inoculation of wells in microplates was prepared as follows. 1 g of soil was weighed, transferred to conical flasks holding 99 cm^3^ sterile peptone water (Buffered Peptone Water, Biocorp^®^) each, and vortexed in an Infors HT Multitron Provortex mixer for 20 min at 130 rpm and at 22°C, after which the samples were cooled for 20 min to 4°C (Pohland and Owen, 2009). Next, 100 mm^3^ was transferred to each of the wells in a FF MicroPlate^®^ and incubated in dark at 28°C for 216 h. The experiment included three replications. The results were read on MicroStation ID systems by the Biolog^®^ company. The extent to which individual carbon sources were used was determined through the reduction of colorless tetrazolium chloride to red formasane (λ = 490 nm) (Islam et al., 2011). Intensity of color development (ICD) was recorded at λ = 490 nm for a period of 216 h at 24-h intervals. The most intensive metabolism of carbon substrates was observed after 48 h of incubation, and therefore the results obtained at that time are presented in the paper. The activity of fungi described in this paper is based on all carbon sources and on grouped sources defined as amines/amides (AN); amino acids (AC); carbohydrate (CB); carboxylic acids (CA); polymers (PY); others (OT). The results were expressed as Average Well-Color Development (AWCD). In addition, the Shannon–Weaver (H) and Substrate Richness (R) indices were calculated from the formulas presented in **Supplementary Table S2**. Finally, four classes of substrate consumption were distinguished, based on OD_490_. They are: OD > 0.75 – high; OD = 0.51–0.75 – good; OD = 0.25–0.50 – medium; OD < 0.25 – low.

### Activity of Dehydrogenases and Catalase

At days 7, 30, 60, 90 and 270, in three replications, samples of unpolluted and polluted soils were submitted to determination of the activity of dehydrogenases with the method described by Öhlinger (1996) and catalase according to the method of Alef and Nannipieri (1998). For each date, a separate series of objects was prepared. At each of the above dates, a series of the experiment was terminated and the whole batch of soil from that series was carefully mixed. This soil served to prepare samples, with three replications, where 6 g were weighed to 25 cm^3^ glass test tubes each for determination of the activity of dehydrogenases, and 2 g of soil were transferred to 100 cm^3^ Erlenmeyer flasks each for determination of the activity of catalase. To determine the activity of dehydrogenases, 60 mg CaCO_3_, 2.5 cm^3^ H_2_O, and 1 cm^3^ of 3% 2,3,5-triphenyltetrazolium chloride (substrate) were added to each test tube with a 6-gm soil sample. Afterward, the samples were incubated an incubator for 24 h in at a temperature of 37°C. Next, triphenylformazan produced during the incubation was extracted from the soil with 96% ethyl alcohol, and the extinction was measured on a Perkin-Elmer Lambda 25 spectrophotometer (Waltham, MA, United States) at a wavelength (λ) equal 485 nm. To determine the activity of catalase, 40 cm^3^ of demineralised H_2_O and 5 cm^3^ of 3% H_2_O_2_ were added to each of the flasks holding 2-gm soil samples. The contents were shaken for 20 min next, an amount of 5 cm^3^ of 0.85 M H_2_SO_4_ was added to each flask and the filtrate was submitted to the determination of the activity of catalase by titration with 0.02 M of potassium permanganate. The activity of dehydrogenases was expressed in μM TFF kg^-1^ d.m. of soil h^-1^, and the activity of catalase in M O_2_ kg^-1^ d.m. of soil h^-1^. By taking into account the activity of dehydrogenases and catalase in unpolluted and polluted soil, the impact factor of diesel oil (IF_DO_) on the activity of soil enzymes was calculated (**Supplementary Table S2**).

### Determination of PAHs

The content of 9 PAHs: naphthalene (NAP), phenanthrene (PHE), anthracene (ANT), fluoranthene (FTH), benzo(a)antracene (BaA), chrysene (CHR), benzo(a)fluoranthene (BaF), benzo(a)pyrene (BaP) and benzo(ghi)perylene (BghiP), was determined in samples of polluted and Ekodiesel Ultra polluted soil samples after 270 days of the experiment (**Supplementary Figure S2**). The accuracy of determination for all PAHs was 0.005 mg kg^-1^, and the recovery yields were form benzo(k)fluoranthene – 87.3% to benzo(a)antracene 120,8%. The content of PAHs was determined on a gas chromatograph Agilent 7890A coupled with a mass spectrometer Agilent 5975C equipped with a source of ions EI. PAHs were determined in WESSLING Company in Cracow, Poland. Hydrocarbons were extracted according to the ISO 18287 standard (ISO 18287, 2006).

### Statistical Analyses

The results were processed statistically in the software program Statistica 12.0 (Statsoft Inc., 2015). Homogeneous groups were identified by using the Tukey’s range test at the significance level *P* = 0.01 and applying analysis of variance ANOVA. The results were also submitted to the PCA (principal component analysis) in order to determine the most intensively metabolized substrates.

## Results

### Activity of Oxireductases and Degradation of PAHs in Soils

**FIGURE 1:**
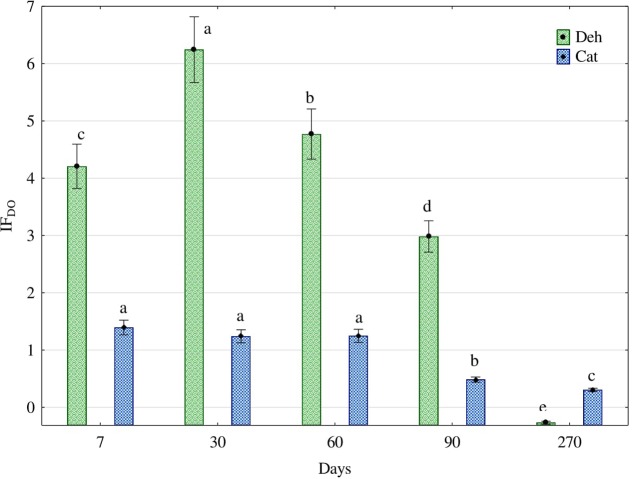
Effect of diesel oil (IF_DO_) on activity of dehydrogenases (Deh) and catalase (Cat). The same letters show homogeneous groups separately for dehydrogenases and separately for catalase. Different letters show significant differences separately for dehydrogenases and separately for catalase (one way ANOVA, Tukey test, *P* = 0.01). Error bars represent standard error of the mean for *n* = 3.

Diesel oil significantly raised the activity of oxireductases in soil (**Figure 1**). Under the influence of diesel oil, the activity of these enzymes was 4.2-fold higher at day 7 after its application, 6.2-fold at day 30, 4.8-fold at day 60 and 3-fold higher at day 90. This trend did not last infinitely long as the activity of dehydrogenases decreased by 0.25-fold at day 270. On the other hand, the activity of catalase in response to diesel oil did not rise so high, ranging from a 1.4-fold increase at day 7 to 0.3-fold at day 270.

**FIGURE 2:**
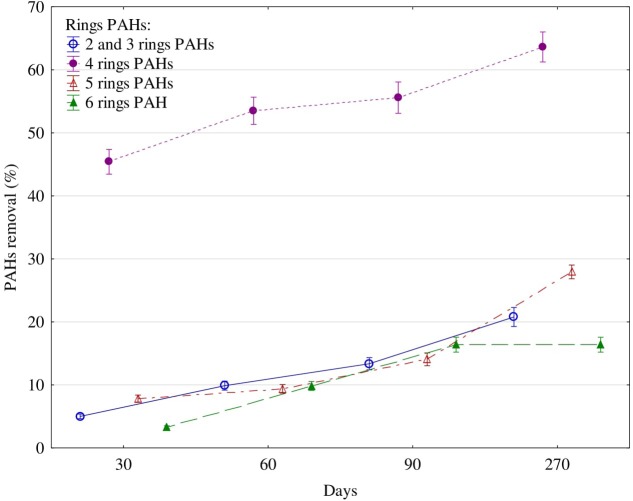
Polycyclic aromatic hydrocarbons (PAHs) removal (%) from soil contaminated with diesel oil after 30, 60, 90, and 270 days. Error bars represent standard error of the mean for *n* = 3.

With time, both the activity of the tested enzymes and the soil content of PAHs decreased (**Figure 2**). Four-ringed hydrocarbons underwent the highest degradation, while six-ringed ones were the least degraded. At day 30, the concentration of four-ringed PAHs decreased by 44%, while the concentrations of the other PAHs were lower by 3–9%. After 270 days, 64% of four-ringed PAHs, 28% of five-ringed, 21% of 2–3-ringed and 16% of six-ringed PAHs had been degraded.

**FIGURE 3:**
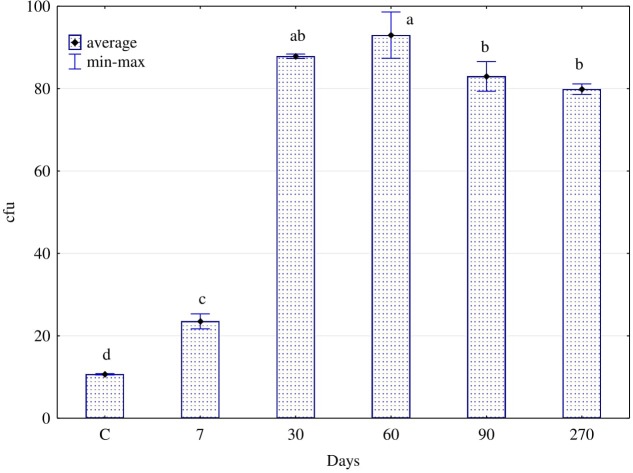
Numbers of fungi (cfu 10^8^ kg^-1^ of d.m. soil) in the soil contaminated with diesel oil after 7, 30, 60, 90, and 270 days (C – control without diesel oil). The same letters show homogeneous groups. Different letters show significant differences between objects for soil fungi (one way ANOVA, Tukey test, *P* = 0.01). Error bars represent standard error of the mean for *n* = 3.

**FIGURE 4:**
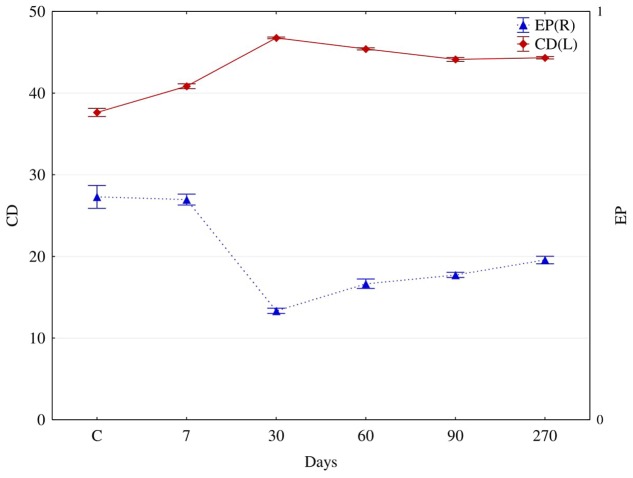
The colony development (CD) and eco-physiological diversity (EP) indices for fungi isolated from soil contaminated with diesel oil (DO) after 7, 30, 60, 90, and 270 days (C – control without diesel oil). Error bars represent standard error of the mean for *n* = 3.

Degradation of hydrocarbons in soil was associated with a higher enzyme activity, which was a consequence of the stimulating effect of diesel oil on the counts (**Figure 3**) and structure (**Figure 4**) of fungi. In soil polluted with diesel oil, the number of fungi was significantly higher than in control soil (**Figure 3**). From days 30 to 270, the number of fungi was stablised in a range from 80⋅10^8^ cfu⋅kg^-1^ of soil dry matter (270 d) to 93⋅10^8^ cfu (60 d).

### Diversity of Fungi in Soil Polluted with Diesel Oil

**FIGURE 5:**
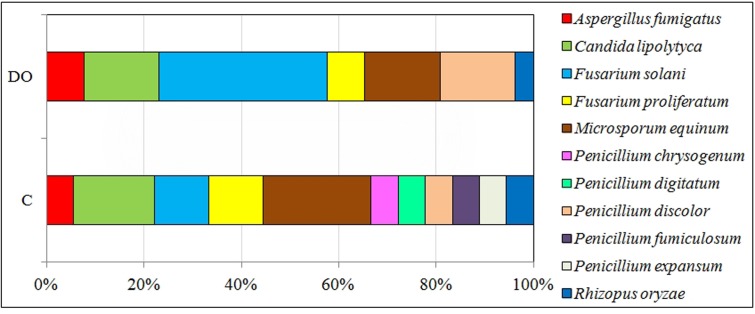
Percentages of fungi identified with the MALDI-TOF method from soil unpolluted (C) and diesel oil polluted (DO).

In diesel oil polluted soil (**Figure 4**), the colony development index (CD) for fungi increased significantly, while their diversity index (EP) decreased. The average value of the CD index for fungi isolated from polluted soil, irrespective of the day of analysis, was 44.3, compared to 37.6 for fungi isolated from control soil. Reversely, the value of EP for fungi isolated from control soil was 0.55, and its average value for fungi from polluted soil, regardless of the day of analysis, was 0.38. Moreover, polluted soil was dominated by fungi from the genera (**Figure 5**): *Fusarium* (37.9%), *Candida* (13.8%), *Microsporum* (13.8%) and *Penicillium* (13.8%), whereas the dominant fungi in control soil were: *Penicillium* (26.5%), *Microsporum* (21.1%), *Fusarium* (21.1%), and *Candida* (15.8%).

### Catabolic Profile of Soil Fungal Communities, Using FF MicroPlates^®^

**FIGURE 6:**
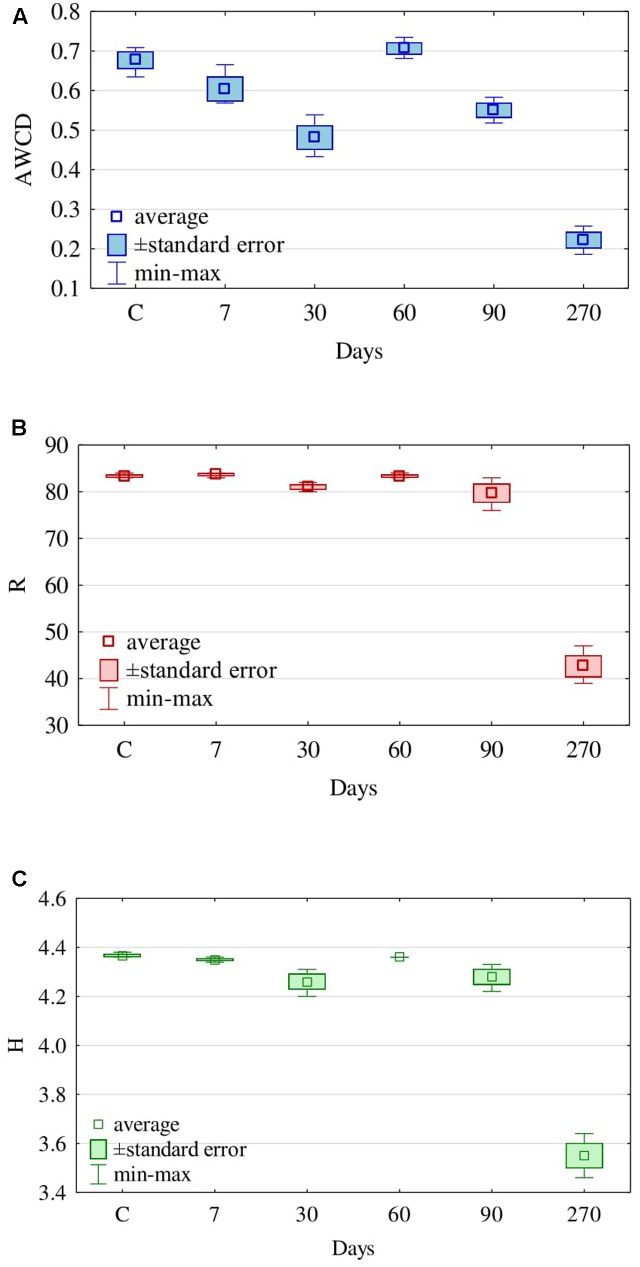
Average **(A)** Well-Color Development (AWCD), **(B)** Richness (R) of carbon substrates and **(C)** Shannon–Weaver index (H) for fungal communities of soil contaminated with diesel oil (DO) using carbon substrates on FF MicroPlates, depending on the duration of soil exposure to diesel oil (C – control without DO).

The retention time of diesel oil in soil had a significant influence on the activity of fungi, although the effects were not unambiguous. The AWCD decreased from day 7 (0.60) to day 30 (0.48), to reach the highest value at day 60 (0.71), while being the lowest at day 270 (**Figure 6A**). Also, the Richness index (R) was the lowest at day 270, when it equalled 43, whereas at the other dates of analyses it was relatively even, ranging from 84 at day 7 to 80 at day 90 (**Figure 6B**). This is reflected by the Shannon–Weaver (H) index of fungi, whose value at day 270 was merely 3.6, fluctuating from 4.3 to 4.4 at the other days of analyses (**Figure 6C**).

The values of these indices depended on the ability of isolated fungi to use various sources of carbon. **Supplementary Table S3** shows that this was a varied capability. 11 sources of carbon were used to a high degree, 53 – to a good degree, 21 – to an average degree and 10 – to a low degree. The best sources of carbon were: Gentobiose (B9), α-Methyl-D-Glucoside (D7), D-Mannitol (D1), D-Melibiose (D4), i-Erythritol (B4), D-Mannose (D2), α-Methyl-D-Galactoside (D5), D-Galactose (B7), D-Glucosamine (B11), L-Fructose (B6) and D-Fructose (B5), whereas the worst ones were: D-Arabinose (A8), Amygdalin (A7), Adonitol (A6), *N*-Acetyl-D-Glucosamine (A4), *N*-Acetyl-D-Galactosamine (A3), L-Arabinose (A9), D-Arabitol (A10), *N*-Acetyl-D-Mannosamine (A5), Tween 80 (A2), and Arbutin (A11).

**FIGURE 7:**
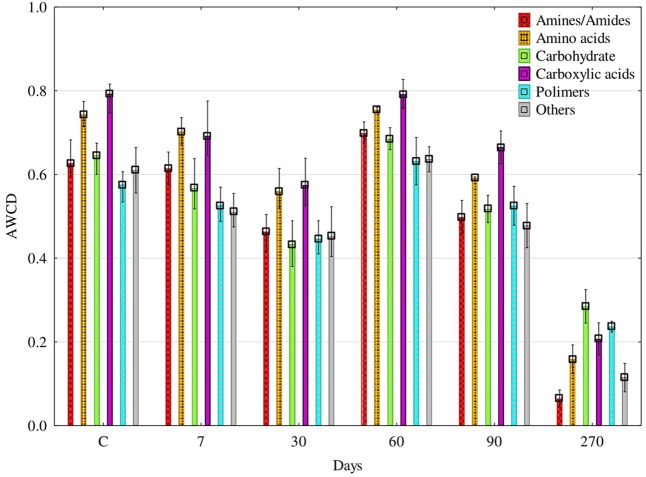
Utilization of different carbon sources groups on the FF MicroPlates^®^ by soil fungal communities depending on the duration of soil exposure to (DO – diesel oil; C – control without DO); AN – amines/amides; AC – amino acids; CB – carbohydrate; CA – carboxylic acids; PY – polymers; OT – others; AWCD – Average Well-Color Development index. Error bars represent standard error of the mean for *n* = 3.

**FIGURE 8:**
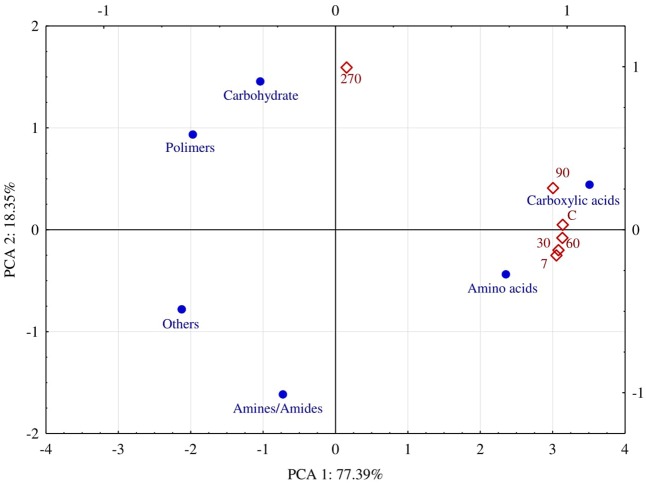
Average Well-Color Development (AWCD) of fungi on FF MicroPlates^®^ and isolated from soil polluted with diesel oil (DO) depending on the duration of soil exposure to diesel oil and carbon sources (C – control without DO, days 7, 30, 60, 90, 270).

The ability of fungi to utilize particular groups of organic compounds was diverse (**Figure 7**). For 90 days, carboxyl acids and amino acids were utilized the best. The AWCD index calculated on the basis of decomposition for the former group of compounds ranged from 0.57 to 0.79, and for the latter – from 0.56 do 0.75. At day 270, the AWCD value decreased to 0.16 and to 0.21, respectively. The PCA results illustrate the above relationships very clearly (**Figure 8**). It is evident that fungi isolated from soil samples contaminated with diesel oil used carbohydrate and polymers as the best sources of carbon, while amines and amides were the poorest sources of carbon. At the other days, carboxyl acids and amino acids were ustilised the best. Utilization of the other groups of organic compounds was much poorer.

## Discussion

One of the most severe threats to the natural environment in central and south-eastern Europe is soil degradation (Günal et al., 2015), in which a large role is played by persistent organic pollutants, including PAHs (Wang et al., 2010; Agnello et al., 2016). Petroleum products which permeate into the soil profile cause changes in the physicochemical properties of soil (Kucharski and Jastrzębska, 2005; Wyszkowska and Kucharski, 2005; Griffiths and Philippot, 2013). In consequence, soil clumping may occur. Thus, petroleum products contribute to the deterioration of physicochemical characteristics of soil (Semrany et al., 2012), which has a negative influence on the growth and development of plants (Sivitskaya and Wyszkowski, 2013; Wyszkowska et al., 2015), but – on the other hand – they are an excellent energy source for certain microorganisms (Kucharski and Jastrzębska, 2005; Wyszkowska and Kucharski, 2005). For these reasons, when describing the quality of soil it does not suffice to determine physical and chemical parameters, even though they are determinants of the growth and development of plants as well as the dynamics of soil metabolism (Aparicio and Costa, 2007; Plaza et al., 2013), but it should be borne in mind that the above paradigm does not account for real-time changes in the soil quality (Lagomarsino et al., 2012; Nannipieri et al., 2012). In our study, the fact that diesel oil could be used by fungi as a source of carbon and energy (Wu et al., 2014; Alrumman et al., 2015; Marchand et al., 2017) probably explained why the number of fungi in DO polluted soil increased and so did the activity of soil oxireductases (**Figure 1**), which are a sensitive indicator of changes in soil quality in real time (Hawrot-Paw et al., 2010; Mohsenzadeh et al., 2012; Kaczyńska et al., 2015). A change in the activity of dehydrogenases and catalase in soil was reflected by the indicators showing the effect of diesel oil on these enzymes. The determination of these indices is fundamental to an objective assessment of the stability of a soil ecosystem exposed to the pressure of a petroleum product. Our experiment lasted for 270 days, which was long enough to observe a tendency for a lowering pressure of the petroleum product tested on dehydrogenases and catalase. However, it should be added that deposition of diesel oil into the soil was performed only once, at the onset of the experiment.

Some petroleum products stimulate the proliferation of fungi (Fan et al., 2014; Silva et al., 2015; El-Hanafy et al., 2017), and bacteria in soil (Sutton et al., 2013; Jiang et al., 2016). Hence, large changes in the activity of the enzymes were determined in soil polluted with diesel oil. For instance, the activity of dehydrogenases increased by as much as 6.2-fold at day 30 of the experiment, while that of catalase was 1.4-fold higher ar day 7 (**Figure 1**), The main reason being the fact that microorganisms are the major source of soil enzymes (Ameen et al., 2016; Stręk and Telesiński, 2016; Zaborowska et al., 2016).

On the other hand, not all hydrocarbons contained in diesel oil were an optimal source of carbon for the community of microorganisms. This most probably stems from the fact that two- and three-ringed PAHs have the highest share in the total polycyclic hydrocarbons (Nganje et al., 2007; Wyszkowska et al., 2015). Also, in this study, throughout the whole experiment, that is 270 days, the prevalent share of all PAHs consisted of 2- and 3-ringed compounds, while six-ringed hydrocarbons made up the smallest proportion (**Supplementary Table S4**). According to Baran et al. (2004), 2- to 4-ringed PAHs are responsible for about 70% of the global PAH contamination. The principal determinants of the diverse composition of PAHs in soil are: the type of pollution caused by petroleum products (Wyszkowski and Ziółkowska, 2013), physicochemical properties of soil (Gałązka et al., 2012) and species of grown plants (Wyszkowski and Ziółkowska, 2013; Nwaichi et al., 2015). In this research, tetravalent hydrocarbons were oxygenated to a higher degree than hexa-, penta-, or di-trivalent ones (**Figure 2**). After 270 days of degradation, the percentage of degraded compounds was 64% of four-ringed, 28% of five-ringned, 21% of 2–3-ringed and 16% of six-ringed PAHs. Alrumman et al. (2015) point to a significant correlation between the degree of degradation of PAHs in soil and the number of fungi This was one of the reasons why the structure and diversity of fungi changed. In polluted soils, the K strategy fungi decreased in number while the r strategy ones became more numerous. Changes in the structure of fungi observed during this study were a response to the biotic stress induced by the pollution with diesel oil. This conclusion is supported by values of the indices CD and EP. The CD and EP indices provide information on changes in ratios between slowly (K-strategists) and rapidly growing microorganisms (r-strategists). The CD index value ranges from 0 to 100. If it approximates 100, a rapid growth of a microbial population in a short time span is implicated. The EP index ranges from 0 to 1, and reflects the evenness of the growth of microorganisms in a given time period. If the value of this index is 1, the growth of microorganisms in a given environment is very uniform (De Leij et al., 1994). The EP index equal 0 suggests a very low functional diversity as all microorganisms grow at the same time. In our study, communities of copiotrophic fungi, quick at responding to the supply of fresh organic matter, including diesel oil, reproduced more intensively. Copiotrophic fungi are essential for efficient utilization of pollution, including contamination with diesel oil. However, they are less representative to individual types and kinds of soils than K-strategy fungi, which can be called oligotrophic fungi. This explains the difference in the dominant species of fungi between the control and DO-polluted soil. The theory of r-strategists and K-strategists presumes that there are genetic differences between microorganisms, which are responsible for the adaptation of fungi to different environments (De Leij et al., 1994). In DO-contaminated soil, some species of fungi can undergo lysis, and are then replaced by new populations of fungi, which are able to degrade PAHs owing to their intensive metabolism (Semrany et al., 2012; Wyszkowska et al., 2015). In unpolluted soil, the dominant species were *Microsporum equinum* and *Candida lipolytica* (**Figure 5**), whereas the dominant species in DO-contaminated soil was *Fusarium solani*, followed by much less dominating species, such as *Candida lipolytica* and *Microsporum equinum*. The differentiated response of autochtonous soil microorganisms to the applied diesel oil could have been caused by the succession of microorganisms (Vazquez et al., 2013; Wu et al., 2014). In the authors’ own studies, no presence of *Penicillium chrysogenum, Penicillium digitatum*, or *Penicillium discolor* was detected in soil exposed to the pressure of diesel oil. In turn, diesel oil was conducive to the development of *Fusarium solani* (**Figure 5**).

Tests on plates from the FF MicroPlate^®^ system by Biolog guarantee obtaining a faithful description of both pure cultures of microorganisms (Mishra and Nautiyal, 2009; Bourdel et al., 2016; Frąc et al., 2016) and their communities in the soil environment (Frąc et al., 2014; Pająk et al., 2016). The functional diversity of fungi isolated from the soil not polluted with DO, determined on FF MicroPlates^®^, was higher than that of fungi isolated from the soil polluted with DO, especially at day 270. This finding is supported by the values (**Figure 6**) of the indices AWCD, substrate Richness (R) oraz Shannon–Weaver (H). There are two reasons behind, one being the depressed ability to use carbon substrates by fungi isolated from polluted soil, and the other consisting of a change in the preference for a carbon source. Soil microorganisms mostly used carboxyl acids and amino acids for the first 90 days and carbohydrates as well as polymers in 270 days (**Figure 8**). The latter can be connected with the need to adapt to the environment. The observation is in accordance with the report of Frąc et al. (2014), who concluded that carbon sources located in the FF MicroPlate^®^ provide a wide range of compounds, which can be used to estimate the functional diversity of soil.

Noteworthy is the coincidence between our evaluation of the functional diversity of fungi identified with the Biolog^®^ system and the assessment of the physiological diversity (EP), based on the culture method carried out on Petri dishes for 10 days. The EP index (**Figure 3**) thus obtained, similarly to the Shannon–Weaver (H) index (**Figure 6C**), was the lowest for fungi isolated from the soil polluted with diesel oil for 270 days.

In conclusion, it is worth underlining that any disturbances in the metabolism of soil and fungal biodiversity should be viewed in the context of soil and plants health and possible recovery of the balance in an ecosystem. This conclusion is in agreement with the European Union Biodiversity Strategy to 2020, which imposes an obligation on all EU member states to make assessments of the condition of soil ecosystems (European Commission, 2014). The initiative is further supported by the Sustainable Development Goals.

## Conclusion

(1) Soil contaminated with diesel oil is characterized by a higher activity of oxireductases and a higher count of fungi than soil not exposed to the pressure of this product.
(2) Soil pollution with diesel oil has an adverse effect on the diversity of fungal populations. This is proved by significantly lower values of the AWCD, Richness Substrates (R) and Shannon–Weaver (H) indices at day 270 after the pollution event.
(3) Effects of diesel oil in soil not submitted to remediation are persistent. After 270, degradation affected only 64% of four-ringed PAHs, 28% – of five-ringed, 21% – 2–3-ringed and 16% of six-ringed PAHs. The long-term effect of DO on fungal communities led to a decline in their functional diversity. This can reduce soil health and grown plants.
(4) The experiment shows that an assessment of the response of fungi to soil pollution with diesel oil made on the basis of colony development on Petri plates (the CD and EP indices) coincides with the results of an analyses performed on microplates (FF MicroPlate by Biolog^®^).

## Author Contributions

AB: conceived and designed the experiments; performed the experiments; isolation and identification of hyphal fungi, determination of: functional diversity of fungi (FF MicroPlates^®^), dehydrogenases and catalase activity, analyzed the data; wrote the manuscript. JW: methodical consultation, participation in the creation of concept the manuscript, verification of the manuscript editorial. KO: participation in the determination of functional diversity of fungi (FF MicroPlates^®^).

## Conflict of Interest Statement

The authors declare that the research was conducted in the absence of any commercial or financial relationships that could be construed as a potential conflict of interest.

## Footnotes

1 www.orlen.pl

## Supplementary Material

FIGURE S1 Spectra for the fungi of soil analysis.

FIGURE S2 Spectra for the PAHs of soil analysis: **(A)** control - soil without diesel oil; **(B)** soil contaminated with diesel oil after 7 days; **(C)** soil contaminated with diesel oil after 30 days; **(D)** soil contaminated with diesel oil after 60 days; **(E)** soil contaminated with diesel oil after 90 days; **(F)** soil contaminated with diesel oil after 270 days.

TABLE S1 General characteristics of experimental soil.

TABLE S2 Used patterns.

TABLE S3 Utilisation of different carbon sources by fungal community incubated on microplates FF MicroPlate^®^ (ICD 490).

TABLE S4 The effect of diesel oil (DO) on the content of polycyclic aromatic hydrocarbons in the soil on day 60 of the experiment, mg PAHs kg^-1^d.m. of soil.
