# Supplementary figures and images for: Functional Diversity of Fungal Communities in Soil Contaminated with Diesel Oil
Source: Front Microbiol. 2017 Sep 27;8:1862. doi: 10.3389/fmicb.2017.01862 (PMC5623761; doi:10.3389/fmicb.2017.01862)

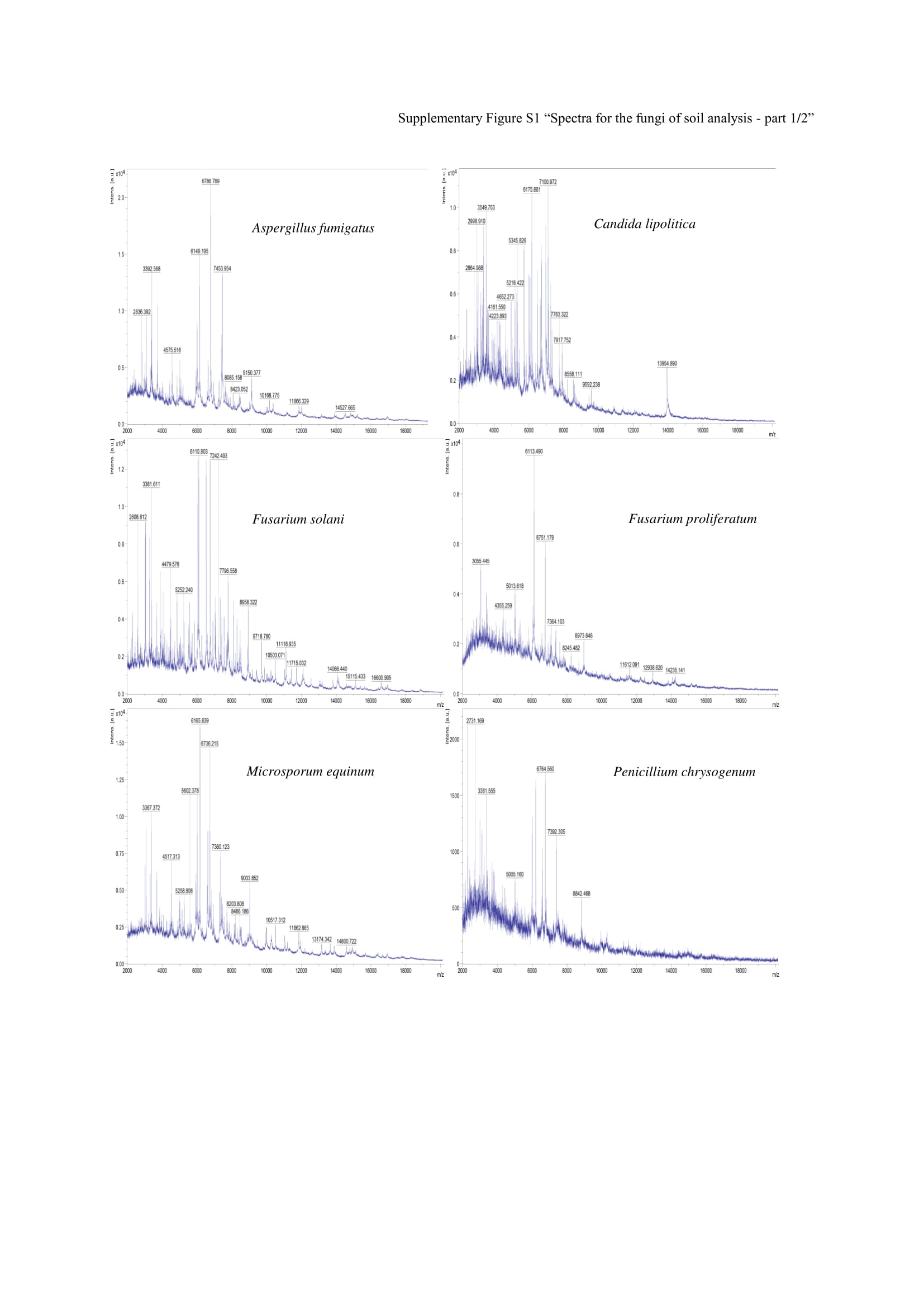

Supplement: FIGURE S1 — Spectra for the fungi of soil analysis. [file Image_1.jpg]

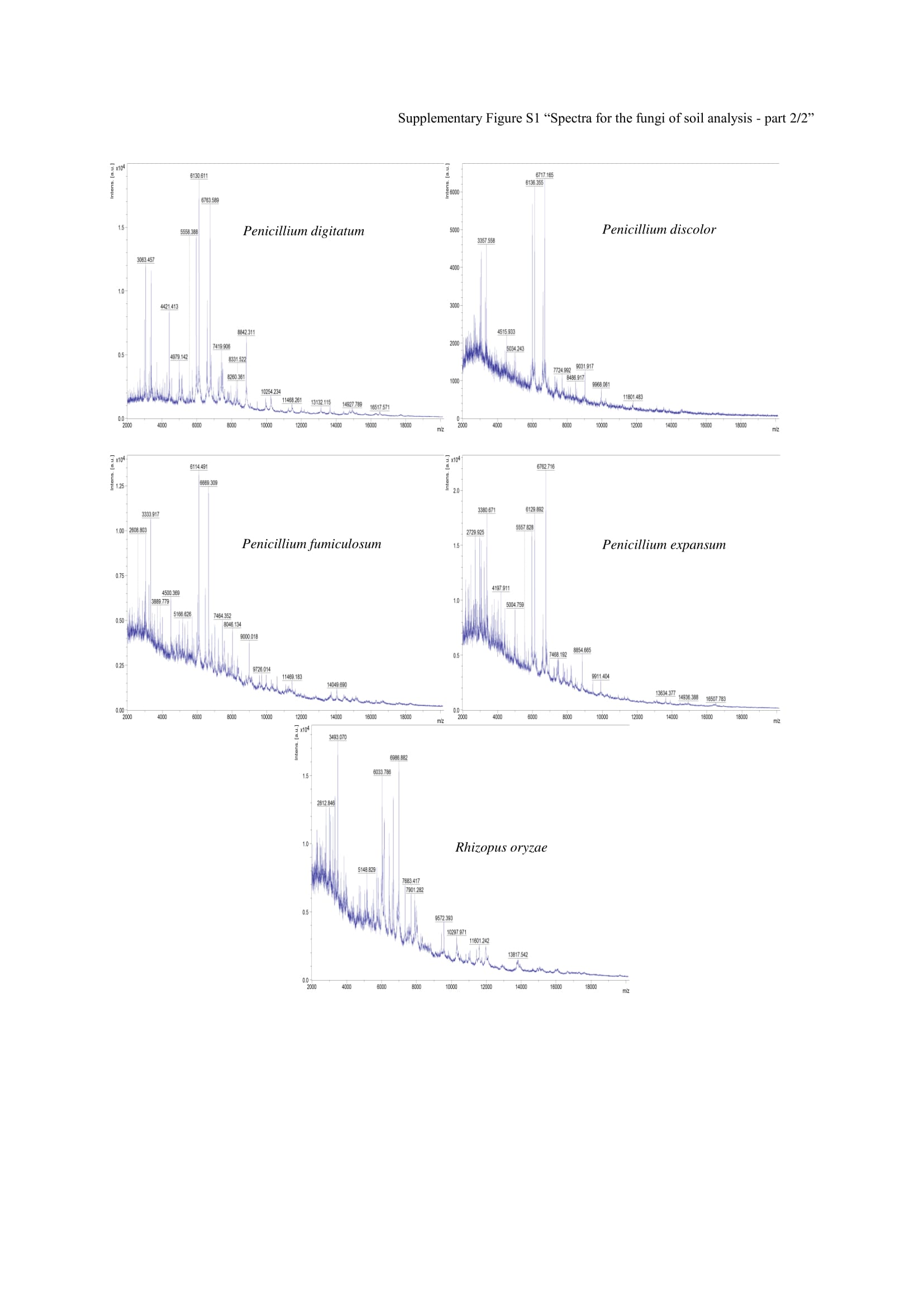

Supplement: FIGURE S2 — Spectra for the PAHs of soil analysis: (A) control - soil without diesel oil; (B) soil contaminated with diesel oil after 7 days; (C) soil contaminated with diesel oil after 30 days; (D) soil contaminated with diesel oil after 60 days; (E) soil contaminated with diesel oil after 90 days; (F) soil contaminated with diesel oil after 270 days. [file Image_2.jpg]

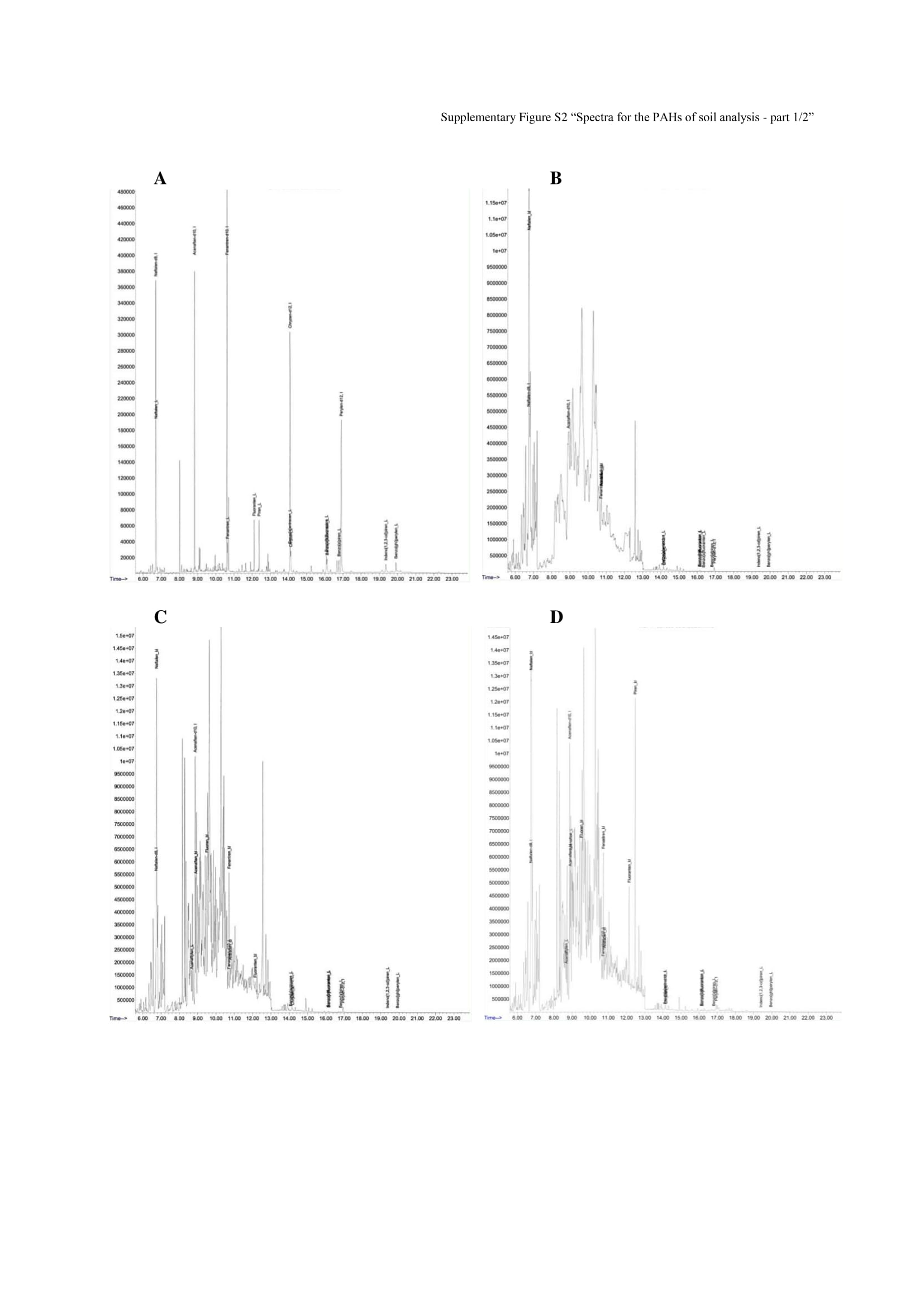

Supplement: Supplementary file 7 [file Image_3.jpg]

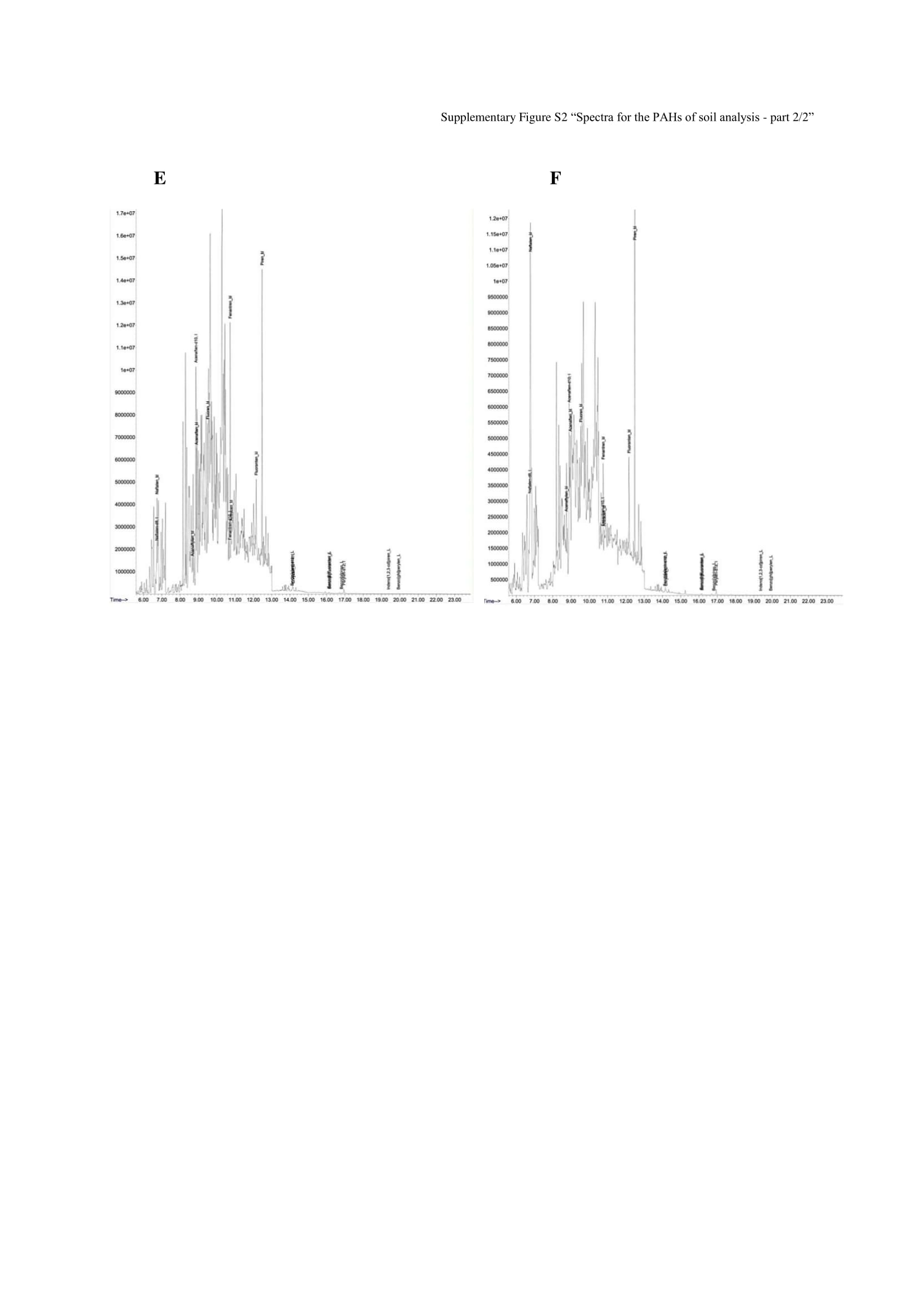

Supplement: Supplementary file 8 [file Image_4.jpg]
