# Supplementary material for: Functional Diversity of Fungal Communities in Soil Contaminated with Diesel Oil
Source: Front Microbiol. 2017 Sep 27;8:1862. doi: 10.3389/fmicb.2017.01862 (PMC5623761; doi:10.3389/fmicb.2017.01862)
Supplement: TABLE S1 — General characteristics of experimental soil. [file Table_1.DOCX]

**Table S1** General characteristics of experimental soil

| Sand | Silt | Clay | C_org_ | N_tot_ | K^+^ | Na^+^ | Ca^2+^ | Mg^2+^ | pH_KCl_ | HAC | EBC | CEC | BS |
| --- | --- | --- | --- | --- | --- | --- | --- | --- | --- | --- | --- | --- | --- |
| Ø µm | | |  |  | exchangeable | | | |  |  |  |  |  |
| 50-2000 | 2-50 | <2 |  |  |  |  |  |  |  |  |  |  |  |
| g kg^-1^ | | | | | mg kg^-1^ | | | |  | mM(+) kg^-1^ | | | % |
| 819 | 167 | 14 | 7.5 | 0.7 | 286 | 80 | 682 | 61 | 5.2 | 18.6 | 39.2 | 57.8 | 67.8 |

C_org_ – organic carbon content, N_total_ – total nitrogen content, HAC - hydrolytic acidity; EBC - exchangeable base cations; CEC - cation exchange capacity; BS - base saturation.
