# Supplementary material for: Functional Diversity of Fungal Communities in Soil Contaminated with Diesel Oil
Source: Front Microbiol. 2017 Sep 27;8:1862. doi: 10.3389/fmicb.2017.01862 (PMC5623761; doi:10.3389/fmicb.2017.01862)
Supplement: TABLE S2 — Used patterns. [file Table_2.DOCX]

**Table S2** Used patterns

| Index | Equation | Explanation | Referencess |
| --- | --- | --- | --- |
| AWCD **–** Average Well-Color Development | AWCD = ∑(C-R)/95 | C is absorbency in each C-source well, R is absorbency in control well | Li et al., 2012  Garland and Mills,  1991; Gomez et al., 2004 |
| H – Shannon-Weaver | H = -Σp_i_(lnp_i_) | pi is the ratio of the absorbency of the each well to the absorbency of all wells | Gomez et al., 2006 |
| R – Substrate Richness | R = C-R | R is the well numbers with (C-R) were calculated using an OD of 0.25 as threshold for positive response | Gomez et al., 2006  Sun et al., 2010 |
| CD – Colony Development | CD = [N1/1 + N2/2 + N3/3….. N10/10] · 100 | N1, N2, N3,...N10 – proportions of microbial colonies identifi ed on days 1, 2, 3,...10. | Sarathchandra et al., 1997 |
| EP - Eco-physiological Diversity | EP = -Σ(pi·log10 pi) | pi – share of individuals of the ith species in the community  relative to the total number of individuals in the community | De Leij et al., 1994 |
| CEC - Sorption Capacity | CEC = EBC + HAC | EBC - exchangeable base cations soil  HAC - hydrolytic acidity soil | Carter, 1993 |
| BS - Base Saturation | BS = (EBC/CEC)·100 | EBC - exchangeable base cations soil  CEC - sorption capacity soil | Carter, 1993 |
| IF_DO_ - diesel oil influence | IF_DO_ = (Po-Co)/Co | Po – enzyme activity in soil contaminated with diesel oil,  Co - enzyme activity in soil without diesel oil | Kaczyńska et al., 2015 |
