# Supplementary material for: Functional Diversity of Fungal Communities in Soil Contaminated with Diesel Oil
Source: Front Microbiol. 2017 Sep 27;8:1862. doi: 10.3389/fmicb.2017.01862 (PMC5623761; doi:10.3389/fmicb.2017.01862)
Supplement: TABLE S3 — Utilisation of different carbon sources by fungal community incubated on microplates FF MicroPlate® (ICD 490). [file Table_3.DOCX]

**Table S3** Utilisation of different carbon sources by fungal community incubated on microplates FF MicroPlate^®^ (ICD 490)

| ICD > 0.75 | | ICD 0.51-0.75 | | | | | | | | ICD 0.25-0.50 | | | | ICD < 0.25 | |
| --- | --- | --- | --- | --- | --- | --- | --- | --- | --- | --- | --- | --- | --- | --- | --- |
| C | ICD | C | ICD | C | ICD | C | ICD | C | ICD | C | ICD | C | ICD | C | ICD |
| *B9* | 0.86 | *D6* | 0.75 | *B3* | 0.68 | *G7* | 0.61 | *E4* | 0.58 | *B1* | 0.50 | *H8* | 0.50 | *A8* | 0.09 |
| *D7* | 0.83 | *D8* | 0.74 | *B8* | 0.67 | *E8* | 0.61 | *G4* | 0.58 | *E2* | 0.49 | *G11* | 0.41 | *A7* | 0.08 |
| *D1* | 0.81 | *F6* | 0.74 | *B10* | 0.66 | *C5* | 0.61 | *E3* | 0.58 | *H9* | 0.49 | *G1* | 0.39 | *A6* | 0.08 |
| *D4* | 0.80 | *D10* | 0.74 | *F7* | 0.66 | *G3* | 0.61 | *E7* | 0.57 | *G12* | 0.49 | *G2* | 0.38 | *A4* | 0.08 |
| *B4* | 0.80 | *H4* | 0.73 | *D11* | 0.66 | *E9* | 0.60 | *H1* | 0.57 | *E1* | 0.48 | *C1* | 0.36 | *A3* | 0.07 |
| *D2* | 0.80 | *D3* | 0.73 | *H5* | 0.64 | *G9* | 0.60 | *H2* | 0.56 | *B12* | 0.47 | *H12* | 0.31 | *A9* | 0.07 |
| *D5* | 0.78 | *H3* | 0.73 | *F1* | 0.64 | *G8* | 0.60 | *D12* | 0.56 | *H11* | 0.46 | *H7* | 0.30 | *A10* | 0.06 |
| *B7* | 0.78 | *D9* | 0.71 | *F9* | 0.64 | *C6* | 0.60 | *G10* | 0.55 | *C8* | 0.46 | *A12* | 0.28 | *A5* | 0.06 |
| *B11* | 0.78 | *F5* | 0.71 | *B2* | 0.62 | *H6* | 0.59 | *E10* | 0.54 | *C2* | 0.46 |  |  | *A2* | 0.05 |
| *B6* | 0.77 | *F8* | 0.70 | *G6* | 0.62 | *F11* | 0.59 | *C12* | 0.54 | *C9* | 0.46 |  |  | *A11* | 0.02 |
| *B5* | 0.77 | *F3* | 0.69 | *F2* | 0.62 | *E5* | 0.59 | *G5* | 0.54 | *H10* | 0.46 |  |  |  |  |
|  |  | *E6* | 0.69 | *C4* | 0.62 | *C7* | 0.59 | *E12* | 0.53 | *E11* | 0.43 |  |  |  |  |
|  |  | *F4* | 0.69 | *C3* | 0.62 | *F10* | 0.58 | *F12* | 0.52 | *C11* | 0.41 |  |  |  |  |
|  |  |  |  |  |  |  |  | *C10* | 0.51 |  |  |  |  |  |  |

C – symbol of carbon source on symbol FF MicroPlates^®^; ICD – intensity of color development

Degree of utilisation of a carbon source: ICD < 0.25 – low; ICD = 0.25-0.50 – medium; ICD = 0.51-0.75 – good; ICD > 0.75 – high.
