# Supplementary material for: Functional Diversity of Fungal Communities in Soil Contaminated with Diesel Oil
Source: Front Microbiol. 2017 Sep 27;8:1862. doi: 10.3389/fmicb.2017.01862 (PMC5623761; doi:10.3389/fmicb.2017.01862)
Supplement: TABLE S4 — The effect of diesel oil (DO) on the content of polycyclic aromatic hydrocarbons in the soil on day 60 of the experiment, mg PAHs kg-1d.m. of soil. [file Table_4.DOCX]

**Table S4** The effect of diesel oil (DO) on the content of polycyclic aromatic hydrocarbons in the soil on day 60 of the experiment, mg PAHs kg^-1^ d.m. of soil

| Days | NAP | ANT | CHR | BaA | DBahA | BaP | BbF | BkP | BghiP | I123cdP | Total PAHs |
| --- | --- | --- | --- | --- | --- | --- | --- | --- | --- | --- | --- |
| C | 0.014  ±0.003 | <0.005 | 0.011  ±0.004 | 0.007  ±0.003 | <0.005 | 0.010  ±0.004 | 0.012  ±0.004 | 0.011  ±0.003 | 0.008  ±0.003 | 0.009  ±0.004 | 0.082  ±0.028 |
| 7 | 13.200  ±3.300 | 1.510  ±0.483 | 0.044  ±0.014 | 0.025  ±0.008 | <0.005 | 0.016  ±0.006 | 0.024  ±0.008 | 0.016  ±0.005 | 0.014  ±0.004 | 0.015  ±0.006 | 14.800  ±3.800 |
| 30 | 13.200  ±3.300 | 0.496  ±0.159 | 0.045  ±0.014 | 0.011  ±0.004 | <0.005 | 0.017  ±0.006 | 0.023  ±0.008 | 0.015  ±0.004 | 0.015  ±0.005 | 0.016  ±0.006 | 13.800  ±3.500 |
| 60 | 13.500  ±3.400 | 0.549  ±0.176 | 0.052  ±0.016 | 0.030  ±0.011 | <0.005 | 0.017  ±0.006 | 0.024  ±0.006 | 0.019  ±0.006 | 0.014  ±0.004 | 0.016  ±0.006 | 14.200  ±3.600 |
| 90 | 12.540  ±0.640 | 1.070  ±0.342 | 0.113  ±0.035 | 0.036  ±0.013 | <0.005 | 0.016  ±0.006 | 0.024  ±0.008 | 0.015  ±0.004 | 0.013  ±0.004 | 0.013  ±0.060 | 13.850  ±1.050 |
| 270 | 11.600  ±2.900 | 0.339  ±0.108 | 0.044  ±0.014 | 0.023  ±0.009 | <0.005 | 0.007  ±0.003 | 0.022  ±0.007 | 0.015  ±0.004 | 0.013  ±0.004 | 0.013  ±0.005 | 12.000±3.050 |

NAP - naphthalene, ANT - anthracene, CHR - chrysene, BaA benzo(a)antracene, DBahA - dibenz(ah)antracene, BaP - benzo(a)pyrene, BbF - benzo(b)fluoranthene, BkF -benzo(k)fluoranthene, BghiP - benzo(ghi)perylene and Indeno(123-c,d)pyrene (I123cdP)
